# The nuts and bolts of excitation–contraction coupling in skeletal muscle

**DOI:** 10.1007/s00421-026-06413-4

**Published:** 2026-09-04

**Authors:** A. F. Dulhunty, M. G. Casarotto

**Affiliations:** 1 John Curtin School of Medical Research, Australian National University, Canberra, ACT Australia; 2 Research School of Biology, Australian National University, Canberra, ACT Australia

**Keywords:** Skeletal muscle, Excitation–contraction coupling, Ca_V_1.1, RyR1, β_1a_ subunit, STAC3, Myopathy

## Abstract

Mobility in humans and all animals depends on the co-ordinated progress of essential steps linking electrical action potentials along the surface membrane of muscle fibres to the increased cytoplasmic Ca^2+^ concentrations that activate the contractile proteins. Excitation–contraction (EC) coupling is the transformation of an electrical signal into a massive release of Ca^2+^ from intracellular Ca^2+^ stores. We consider the evolution of our knowledge of the structures and proteins that underlie skeletal muscle EC coupling, unanswered questions and future developments that will reveal the fundamental molecular events of the coupling process. The deeper understanding gained with future developments will reveal the molecular basis of muscle weakness related to mutations in EC coupling proteins or result from fatigue and aging. The benefits will flow on to the rational design of therapies to increase muscle strength in the many situations where muscle weakness is related to changes in EC coupling.

**Graphical Abstract:**

**Figure:**
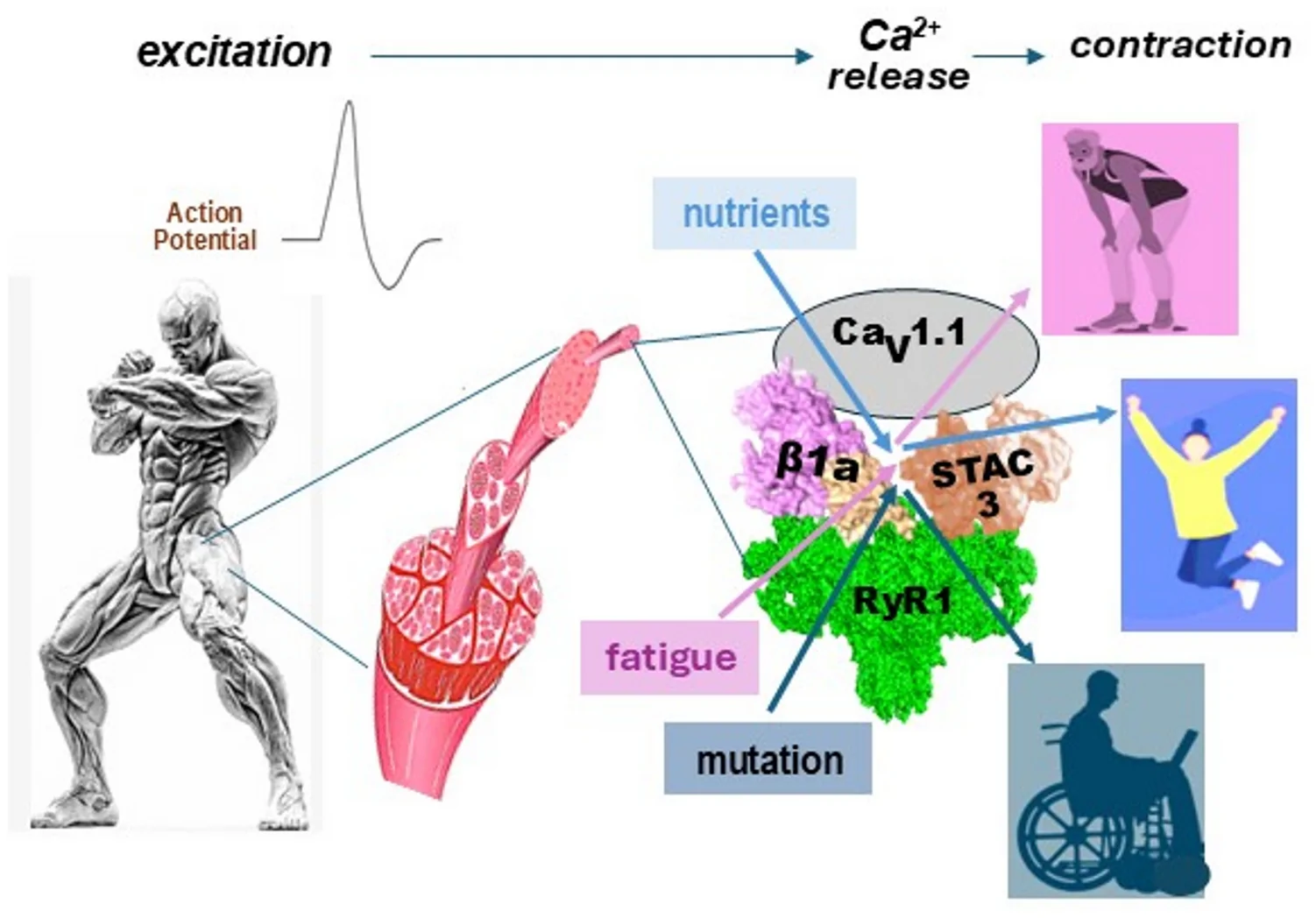

## Introduction

Excitation–Contraction coupling (EC coupling) is an essential step in the control of muscle contraction by action potential signals which are normally initiated in the central nervous system (CNS). The term EC coupling can be used to describe the overall process linking the action potential on the surface of a muscle fibre to contraction, although it is generally used to more specifically describe the transmission of the signal from the surface membrane to the intracellular membrane surrounding the sarcoplasmic reticulum (SR) Ca^2+^ store to initiate the release of the Ca^2+^ that activates the contractile proteins. In skeletal muscle this process depends in an intimate physical interaction (i.e. mechanical coupling) between four proteins that span the junction between the surface and intracellular membrane systems, namely Ca_V_1.1 and its beta subunit (β_1a_), STAC3 and RyR1. A characteristic of skeletal muscle is that contraction force is finely tuned by intracellular factors which can impact the contractile proteins either directly or indirectly by modifying the function of the proteins supporting EC coupling. Overall, Ca^2+^ homeostasis reflects an integration of the amounts of Ca^2+^ released from the SR with the metabolic capacity of the muscle fibre as well as the metabolic and mental capacity of the animal. Ca^2+^ release from the SR depends on the cytoplasmic concentrations of various soluble factors including Ca^2+^, Mg^2+^, K^+^, ATP, H^+^ and inorganic phosphate all of which are altered by various factors including exercise and muscle fatigue (Cairns and Lindinger 2025; Allen et al. 2008). These factors directly modulate Ca_V_1.1 and RyR1 opening during EC coupling (O'Connell et al. 2002; Nayak et al. 2024; Yuan et al. 2021). Their effects on the function of the other proteins in the EC coupling pathway are largely unknown. Muscle strength and mobility can be severely reduced by congenital myopathies in RyR1 (Wilmshurst et al. 2010) and in Ca_V_1.1, β_1a_ or STAC3 (Perez et al. 2018; Wong King Yuen et al. 2017; Iyer et al. 2020). Finally changes in EC coupling contribute muscle weakness in aging (Delbono 2011). In many of these situations the strength of contraction can be increased by drugs like caffeine that bind directly to the RyR1 to enhance Ca^2+^ release (Chirasani et al. 2021). Malignant Hyperthermia mutations in RyR1 lead to excess Ca^2+^ release which is supressed when the drug dantrolene binds to RyR1 (Gaburjakova and Gaburjakova 2023). Understanding of the molecular processes that underly EC coupling will lead to the rational design of drugs to alleviate detrimental changes in EC coupling and will depend critically on our knowledge of the high-resolution structure of the proteins and mechanisms that support EC coupling.

EC coupling is fundamentally different in the heart where the coupling depends on the diffusion of Ca^2+^ ions from the cardiac Ca_V_1.2 to the cardiac Ca^2+^ release channel (RyR2). Ca_V_1.2 and RyR2 are located mainly at diadic junctions between the exterior or T-tubule membranes and the SR, in contrast to the triadic junctions in skeletal muscle (Franzini-Armstrong 2018). RyR2 is activated by the Ca^2+^ ions that enter the myocyte through Ca_V_1.2. None of core skeletal EC coupling proteins (Ca_V_1.1, RyR1, β_1a_ or STAC3) are expressed in the heart (Nakai et al. 1997, 1998; Grabner et al. 1999; Schredelseker et al. 2009; Flucher and Campiglio 2019). Importantly, Ca_V_1.2 and RyR2 are unable to support skeletal EC coupling if expressed in skeletal myocytes or myotubes (Nakai et al. 1997, 1998; Grabner et al. 1999; Schredelseker et al. 2009). The challenges with understanding EC coupling in the heart concern geometrical aspects of the diffusion of Ca^2+^ from Ca_V_1.2 to RyR2 (Eisner et al. 2017) and will not be considered further here where we focus on the very different challenges of understanding mechanical EC coupling in skeletal muscle.

The nuts and bolts of skeletal muscle EC coupling have occupied the minds of muscle physiologists worldwide for many years. In this communication we provide an overview of progress in our understanding of skeletal EC coupling from early observations published in the late 1940’s to the present time. The historical perspective is used to illustrate the monumental conceptual leaps over the years and to underline the extent of detailed information supporting the present state of the field. We introduce the major proteins RyR1, Ca_V_1.1, β_1a_, and STAC3 and discuss aspects of each that are relevant to their roles in skeletal EC coupling. A more detailed discussion of the structure of the membrane skeleton surrounding and supporting the proteins to allow their physical interactions is then provided followed by a more detailed consideration of the structures of the individual proteins and potential protein/protein interaction sites. We end with a discussion about recent emerging developments in in-situ cryo-electron tomography (cryo-ET) techniques (Xu et al. 2024) and speculation about ways that resolution of the images may be improved by combining data from high resolution cryo-EM with AlphaFold-Multimer predictions of the tetrad complex, molecular dynamics simulations, and the FRET-based reporters to develop a higher resolution understanding of the molecular structural dynamics within the junctional region during EC coupling. The immediate benefit of these results will be a greater understanding of changes that occur within the triad junction when EC coupling and Ca^2+^ release from the SR is depressed and underpin the future design of more effective therapeutics to boost Ca^2+^ release. A goal for the more distant future is that in addition to the currently static images obtained with in-situ cryo-ET, novel techniques might be developed allow real time imaging of molecular changes occurring during EC coupling in living muscle fibres.

### The evolution of our present understanding the EC coupling mechanism

**Fig. 1:**
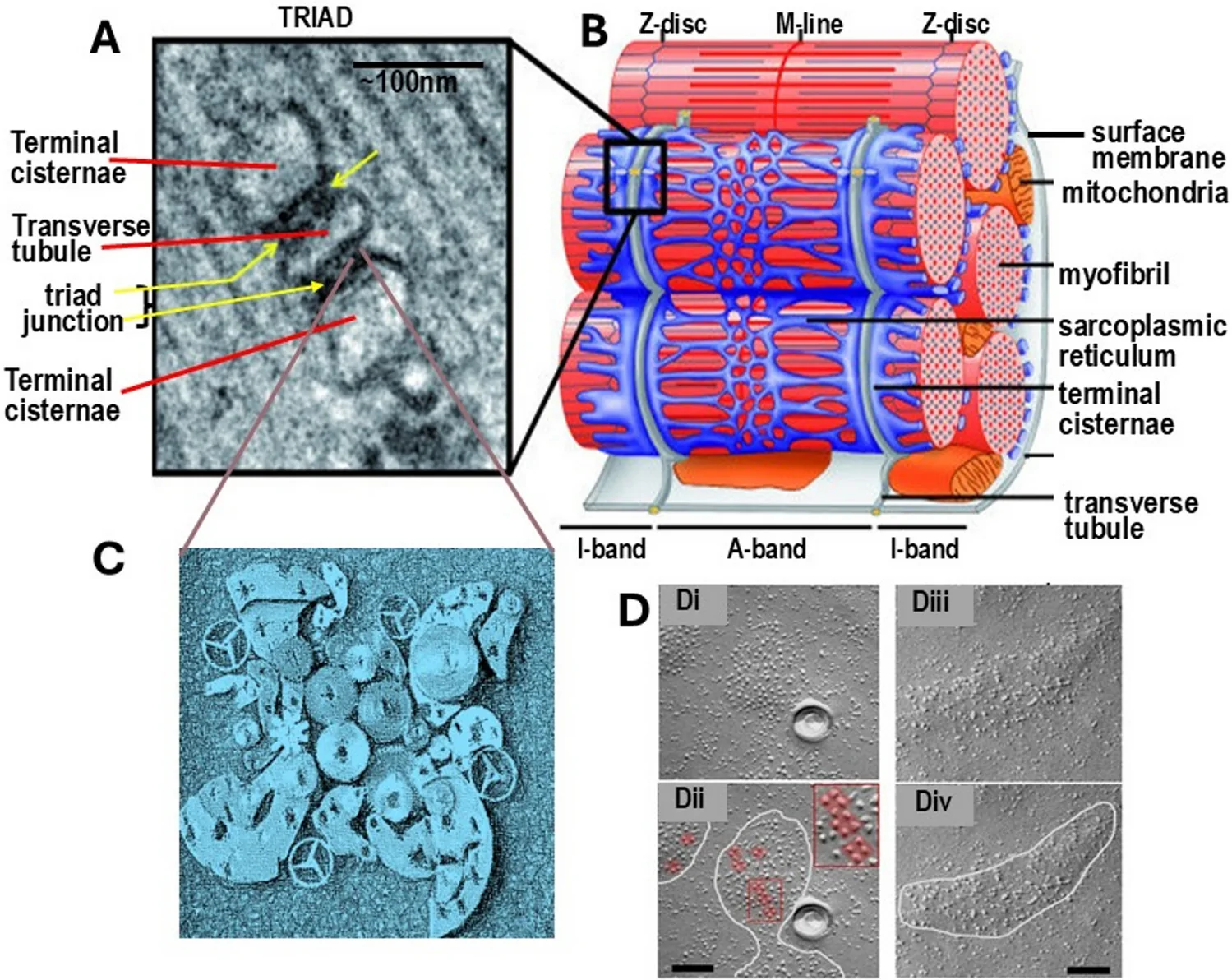
Subcellular location and arrangement of the EC coupling machinery in the triad junction of mammalian skeletal muscle fibres and muscle-derived cell lines. **A**, **B** The triad junction. Modified from Al-Qusairi and Laporte (Al-Qusairi and Laporte 2011). **A** Electron micrograph of a triad junction showing the terminal cisternae (TC) of the SR on either side of a T-tubule with the junctional gap between two membranes on either side of the T-tubule, indicated by the yellow arrows. The red lines point to the TC and the lumen of the T-tubule as labelled. Electron densities in the junction between the T-tubule and TC indicate the position of the DHPR/RyR1 multi-protein complex. **B** Schematic arrangement the triad complex wrapping around myofibrillar bundles. **C** Mechanical representation of the complexity of DHPR/RyR1 multi-protein complex in an orientation parallel with the junctional face membrane, at 90° to the section shown in (**B**). The image in **C** is modified from an artistic interpretation credited to Dr. Montserrat Samsó from the SBGrid Consortium. **D** Freeze fracture replicas of the plasma membrane of tsA201 cells, modified from (Perni et al. 2017). The replica in (Di) and (Dii) shows tetrad formation in a cell where all 5 proteins (RyR1, Ca(V)1.1, β_1a_, STAC3 and junctophilin2) are expressed. Freeze fracture replicas show fewer, if any, tetrads in junctions if any of the four core proteins are not expressed. The replica in Dii and Div lacking tetrads is from a cell expressing Ca(V)1.1, DHPR β_1a_, StAC3 and junctophilin2, but not RyR1. In the micrographs in (Dii) and (Div)**,** the broken white lines surround domed regions overlying junctions between the endoplasmic reticulum and plasma membrane. Tetrads are apparent in (Dii) as clusters of four neighbouring large particles within a 30 nm x 30 nm square area that reflects the dimensions of the RyR1 tetramer. The tetrad formation indicates a physical connection between Ca_V_1.1 and RyR1. In (Dii) Particles in tetrads in are marked with red dots. The tetrads are magnified ×2 in the inset in (Di). The scale bars in (Dii) and (Div) are 100 nm

Investigations into the nature of EC coupling arguably began with AV Hill’s inciteful prediction in 1949 that there must be an action potential conduction pathway throughout the muscle fibre in order to explain the rapid contraction of skeletal muscle following action potential stimulation (Hill 1949). The conduction pathway, via the T-tubule extensions of the surface membrane, was discovered by early electron microscopists in the 1960s when the triad junction between the T-tubule and the SR Ca^2+^ store was recognised as the likely site of signal transduction (Fig. 1) (Franzini-Armstrong and Porter 1964; Franzini-Armstrong 1970). It was initially thought coupling depended on the diffusion of an ion or soluble transmitter across the junction. However evidence that the coupling is mechanical (or physical), rather than diffusion-based was provided by Schneider and Chandler in 1973 (Schneider and Chandler 1973). Identification of the molecular components of EC coupling began with the two membrane-bound ion channel proteins. The Ca^2+^ release channel embedded in the SR membrane was the first, being identified as the skeletal isoform of the giant 2.4MDa RyR1 (Meissner 1986; Inui et al. 1987; Lai et al. 1987). Then the voltage-sensor in the T-tubule membrane was identified as Ca_V_1.1 (then known as the dihydropyridine receptor, DHPR) (Lamb 1987; Rios and Brum 1987; Takekura et al. 1994). These proteins alone could not account for all the characteristics of skeletal muscle EC coupling and the search began for coupling proteins. In the following years the soluble β-subunit of Ca_V_1.1 (β_1a_**)** and STAC3 were found to be additional essential components of signal transmission across the triad junction (Gregg et al. 1996; Horstick et al. 2013). It was later established that expression of five proteins, Ca_V_1.1, β_1a_, STAC3, RyR1 and junctophilin 2 form the minimal requirement to reconstitute mechanical coupling in a muscle-derived cell line (Perni et al. 2017). Of these, junctophilin plays a mainly structural role by spanning the junctional gap between Ca_V_1.1 and RyR1 and maintaining the correct geometry of the junction to enable the other components of EC coupling to interact appropriately to transmit the EC coupling signal (Ito et al. 2001). The other four proteins presumably constitute the transmission pathway from the T-tubule to the SR. β_1a_ and STAC3 have additional roles in facilitating the correct post-translational trafficking of the partner proteins into the correct cellular location to contribute to EC coupling (Campiglio et al. 2018; Bichet et al. 2000; Gerster et al. 1999). Collectively Ca_V_1.1, β_1a_, STAC3, and RyR1 will be referred to as the “core EC coupling proteins”.

To summarise, since the late 1940s the four core proteins required for skeletal EC coupling have been identified and their functional and structural characteristics extensively examined. Binding sites that might allow the proteins to interact with each other during transmission of the coupling signal have been identified. The high-resolution structures of the essential proteins have been determined (Iyer et al. 2020; Woll et al. 2021; Wu et al. 2016; Wong King Yuen et al. 2017). Our future challenges will be focussed at understanding the fundamental molecular changes within and between the proteins that transmit the EC coupling signal to the SR to release the Ca^2+^ ions required for activation of the contractile proteins. The known facts about each component of the skeletal EC coupling pathway are summarised in the following sections.

### The structural framework for EC coupling within skeletal muscle fibres: the T-tubule-SR junction

T-tubules are tiny invaginations of the surface membrane of skeletal muscle fibres, arising mainly from surface membrane caveolae (Dulhunty and Franzini-Armstrong 1975) and traversing the entire cross-section of the fibre, forming triadic junctions with the terminal cisternae (TC) of the SR along the way (Fig. 1A) (Franzini-Armstrong 1970; Al-Qusairi and Laporte 2011). The T-tubules and triad junctions are strictly aligned with the myofilament arrangement, located at the Z-line in amphibian muscle or junction of the A and I band in mammalian muscle (Fig. 1B). Longitudinal extensions between T-tubules in adjacent sarcomeres were described at the same time (Franzini-Armstrong 2018). The longitudinal tubules were later found to be important in conducting action potentials longitudinally inside muscle fibres and around adjacent sarcomeres (Posterino et al. 2000; Edwards et al. 2012). It is notable that the structure and dimensions of the triad junction including the T-tubule membrane, the junctional gap and SR membrane (Fig. 1A), are identical across a wide variety of healthy skeletal muscle fibres, with contraction speeds and T-tubule/SR geometries that can substantially vary between species, muscle type and fibre type (Franzini-Armstrong 2018). The width of the tiny cytoplasmic junctional gap between the T-tubule and SR membranes apparent in electron micrographs in Fig. 1A is similar to the width of the bordering membranes, i.e. ~9-10 nm. The constant separation between the membranes suggests that the arrangement of the proteins within the junction is essential for the normal EC coupling. Indeed, changes in the junction with deletion or modification of the essential proteins is invariably associated with significant changes in EC coupling, for example Takekura et al. (1995).

### Essential proteins – the nuts and bolts of skeletal EC coupling

**Fig. 2:**
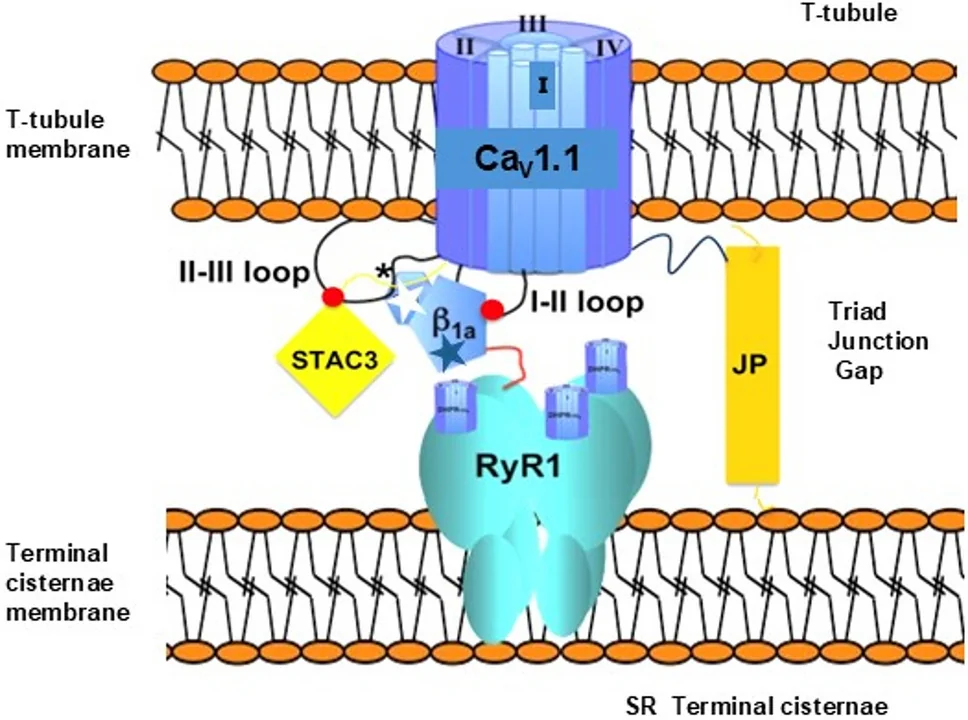
The five essential proteins for skeletal EC coupling**.** The four core EC coupling proteins (Ca_V_1.1, β_1a_, STAC3 and RyR1) and junctophilin2 are shown in a possible binding arrangement between the T-tubule and SR membranes. One subunit of the RyR1 ion channel tetramer is shown potentially linked to the II-III loop of one member of a Ca_V_1.1 tetrad via β_1a_ and STAC3. The red dots indicate known interactions between the Ca_V_1.1 I-II loop with the β_1a_ GK domain and between the Ca_V_1.1 II-III loop and the SH3 domain. The black asterisk indicates a postulated binding site between the Ca_V_1.1 II-III loop and the β_1a_ SH3 domain. The large white star covers the SH3 domain of β_1a_. The large navy-blue star is in the GK domain of β_1a_. The red line depicts a potential interaction between the β_1a_ C-terminal tail and RyR1. The proximal C-terminus of Ca_V_1.1 binding to C1 and linker of STAC3 (Fig. 5 below) is not shown for simplicity. Modified from (Karunasekara et al. 2025)

Although the four core EC coupling proteins, Ca_V_1.1, β_1a_, STAC3 and RyR1 are sufficient to reconstitute mechanical coupling into muscle derived cell lines, many other soluble and membrane bound proteins help maintain the structure of the junction and can modulate the coupling process (Perni et al. 2017). The mechanical model in Fig. 1C emphasises the interconnected arrangement of the core EC coupling proteins within the junctional gap and the multiple potential interactions between the proteins. The model reflects the likely fourfold symmetry of the protein complex based on the homo-tetrameric structure of the Ca^2+^ release channel. That such a model is appropriate emphasises the fact that we still don’t know the precise arrangement of the proteins that associate with RyR1 within the 30 nm x 30 nm cross sectional area encompassing its cytoplasmic domain. It has been established that every second RyR1 channel is coupled to four Ca_V_1.1 molecules, and that the alternating RyR1 channels are not physically coupled to Ca_V_1.1 (Block et al. 1988; Franzini-Armstrong 2018). Each coupled RyR1 tetramer is associated with four Ca_V_1.1 molecules that form a particle cluster termed a tetrad in freeze-fracture images of the junctional T-tubule membrane (Block et al. 1988) and plasma membrane of cell lines expressing RyR1, Ca_V_1.1, DHPR β1a, Stac3, and junctophilin2 (Fig. 1D) (Perni et al. 2017). The dimensions of the tetradic cluster closely mimic the dimensions of the RyR1 tetramer (Fig. 1D). The tetrad structure is essential for normal EC coupling and the structure is disrupted with mutations in any of the core EC coupling proteins that impact on normal skeletal EC coupling (Takekura et al. 1994). Notably tetrad particle arrangements are not seen in freeze-fracture replicas of T-tubule/SR junctions of cardiac myocytes (Asghari et al. 2020), consistent with cardiac EC coupling depending simply on diffusion of Ca^2+^ ions from Ca_V_1.2 to RyR2. The dependence of skeletal EC coupling on tetrad structure is indicated by numerous reports that deletion of the Ca_V_1.1 II-III loop as well as the absence of β_1a_ or STAC3 disrupt both skeletal EC coupling and tetrad formation (Takekura et al. 2004; Dayal et al. 2022; Linsley et al. 2017). These observations, in addition to the absence of definitive evidence showing direct binding of CaV1.1 to RyR1 in vitro*,* strongly support the concept of a physical coupling between the membrane proteins via the essential cytoplasmic proteins (Fig. 2).

There are approximately twice as many Ca_V_1.1 voltage-sensor proteins as RyR1 tetrameric ion channels in the triad complex. There are four Ca_V_1.1 per RyR1 ion channel tetramer for couped RyR1 channels or 2:1 if both coupled and uncoupled RyR1 tetramers in junctional arrays are included. The precise relationship between RyR1 and Ca_V_1.1 and β_1a_ or STAC3 is less clear. It is a reasonable assumption that each alpha Ca_V_1.1 molecule is associated with one β_1a_ subunit and with one STAC3 molecule (Fig. 2). However, the exact numbers are unknown and transient interactions between the molecules are possible, as suggested with FKBP12 and RyR1 (Richardson et al. 2025), so that the exact relationship between the molecules is unknown at any one time in individual triad junctions. On the other hand, as mentioned, there is overwhelming evidence the four proteins interact physically with each other to allow full strength EC coupling (Perni et al. 2017). The challenge now is to understand the arrangement of the components in the triad junction and just how these interactions proceed to facilitate contractile function in a living muscle fibre.

The question that arises from the observation that half of the RyR1 tetramers in the triad junction are not coupled to Ca_V_1.1 tetrads concerns the way that the uncoupled RyR1 channels are activated during EC coupling. The possibility that the uncoupled channels are activated by Ca^2+^ released through coupled RyRs has not been excluded, while a viable alternative mechanism is a physical communication with Ca_V_1.1-coupled RyR1 channels (Meissner 2017). Indeed, potential interaction sites have been identified (Xu et al. 2024). Furthermore, evidence for physical coupling is provided by RyR1 channels in lipid bilayer exhibiting “coupled gating” where two or more channels can open simultaneously (Marx et al. 1998; Dulhunty et al. 2018).

### The Ca^2+^ release channel in the junctional face membrane of sarcoplasmic reticulum

**Fig. 3:**
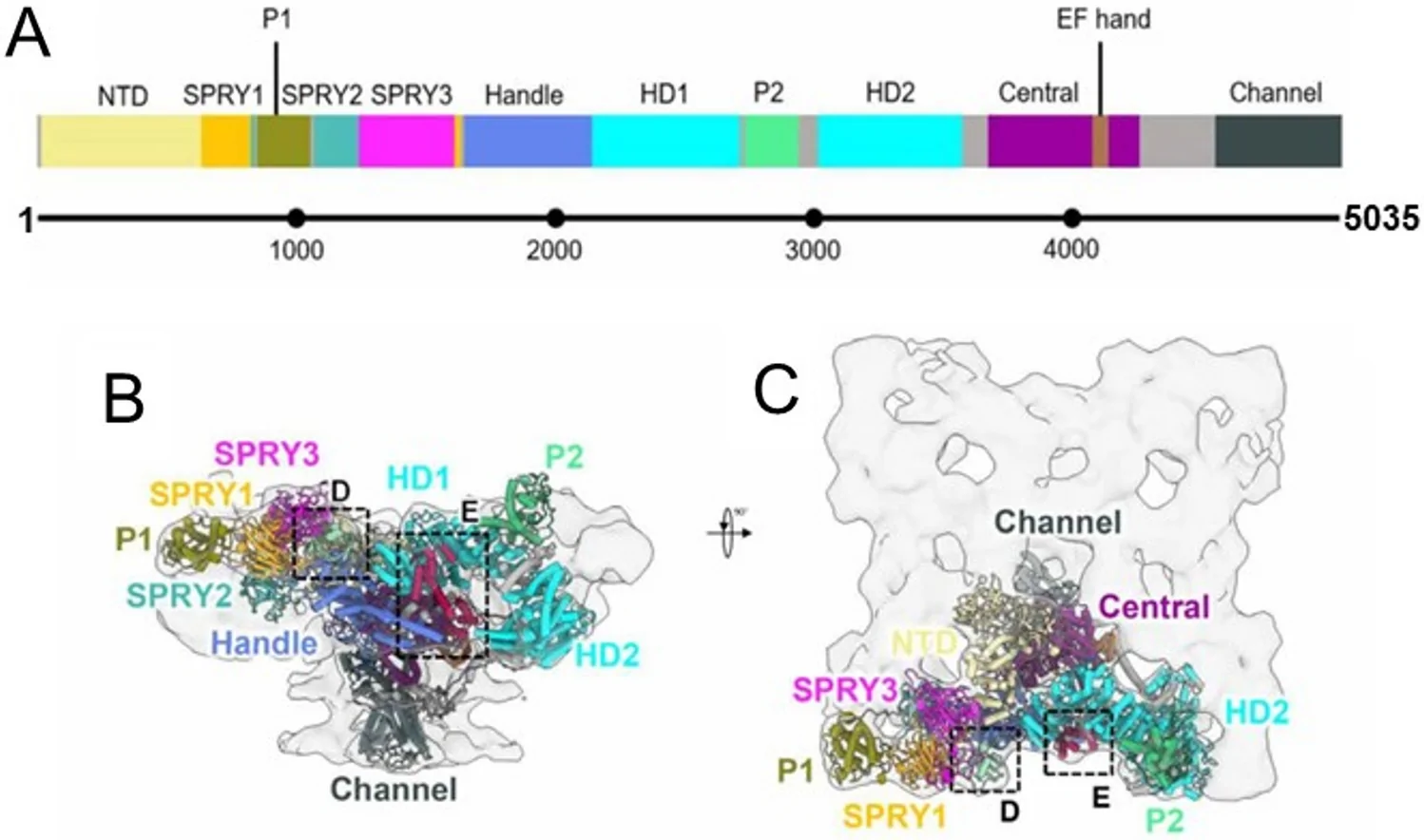
The structure of RyR1**. A** Linear sequence showing colour coded larger structural domains of RyR1. **B**, **C** Cryo-EM structures of RyR1. The RyR1 domains are colour coded in the same way as in (**A**) and have been fitted into the cryo-EM maps of adjacent monomers of the ion channel tetramer which is oriented with the cytoplasmic domain facing the T-tubule membrane at the top of the structure. The ion channel pore at the base of the structure is largely embedded in the TC membrane and enters the lumen of the SR. **B** a cross-section through the cytoplasmic domain of RyR1 channel. The image is oriented at right-angles to the plane of the SR and T-tubule membrane with sections through two of the four monomers that form the ion channel tetramer. The sections through the monomers are offset to illustrate the positions of adjacent domains that exist within each monomer. **C** a cross-section through the cytoplasmic domain of the RyR1 channel at right-angles to the plane of the SR membrane, revealing all four monomers surrounding the central cavity leading to ion channel pore. The broken lines **B** and **C** surround boxes labelled D and E which contain the FKBP12 region and apo-CaM binding regions respectively. Modified from (Xu et al. 2024)

RyR1 channels are located in a crystalline-like array in the SR membrane that forms the intracellular boundary of the triad junction and is termed the “junctional face membrane” (Franzini-Armstrong 2018). A “ryanodine receptor” of some form was thought to be involved in EC coupling in the 1980s due to the sensitivity of the EC coupling process to the herbicide ryanodine (Meissner 1986; Inui et al. 1987). The high molecular weight RyR1 protein was cloned and sequenced in 1989 (Takeshima et al. 1989) (Fig. 3A). Three isoforms of the RyR were identified. RyR1 mainly in skeletal muscle, RyR2 mainly in the heart and brain and RyR3 initially found in brain but then in many other tissues. Definitive evidence that RyR1 was in fact the Ca^2+^ release channel in skeletal muscle came in the early 1990’s when EC coupling was abolished after RyR1 knockout and restored when recombinant RyR1 was expressed in RyR1-null myocytes (Moore et al. 1998) and myotubes (Avila and Dirksen 2000).

The characteristics of the opening of single RyR1 ion channels have been extensively examined in a variety of isolated preparations. The literature has been summarised in reviews such as (Meissner 2017; Dulhunty et al. 2018) and will not be considered further here. The remainder of this section provides a summary of studies leading to our current knowledge of the structure of the RyR1 protein. Excellent detailed reviews of these early structural studies are available and are recommended reading (Franzini-Armstrong 2018; Meissner 2017; Shishmarev 2020). Briefly, the RyR1 protein was clearly visible in the earliest thin section electron micrographs of skeletal muscle (Fig. 1A). Within the triad junction the RyR1 channels form arrays of electron dense material or “junctional feet” spanning the gap between T-tubule and SR membranes at T-tubule/SR junctions (Franzini-Armstrong 1970). Rotary shadowing revealed that each foot had a regular four-fold structure (Ferguson et al. 1984) and the foot protein was identified shortly thereafter as the homo-tetrameric RyR1 Ca^2+^ release channel (Lai et al. 1987).

The size of the RyR1 ion channel tetramer has provided significant challenges for determining its high-resolution structure. Early cryo-EM structures with a resolution of ~30A^o^ (Wagenknecht et al. 1989) confirmed that the ion channel was formed by four RyR1 monomers adopting an overall mushroom shape with the cap occupying the cytoplasm in the T-tubule/SR junction, a stalk crossing the SR membrane and a very small domain entering the SR lumen. The resolution of cryo-EM images increased progressively over the next 20 years to ∼10 Å, allowing mapping of structural changes in well-defined domains associated with channel opening and closing (Ludtke et al. 2005; Samso et al. 2005, 2009). The quality of the images gradually improved further with resolution increasing to 3.6 to 6.2 Å (Meissner 2017) and most recently to levels approaching atomic resolution of ~3.0 Å in some highly structured regions (Iyer et al. 2020; Woll et al. 2021) (Fig. 3B, C). Developments in both electron microscopes and advances in computational analysis techniques have allowed developments during the past 10 years that were previously unimaginable. It is worth noting that the high-resolution structures of very large proteins such as the RyR tetramer have often been limited to specific regions having more rigid structure while the structures of flexible regions remained unresolved. Use of X-Ray crystallographic and Nuclear Magnetic Resonance (NMR) methods have been adopted and have provided a complementary approach that has yielded unique and valuable structural information not provided by cryo-EM alone (Kimura et al. 2009; Lau and Van Petegem 2014). Cryo-EM techniques have now evolved to the stage where changes in the orientation of individual amino acid residues throughout RyR1 tetramer can be followed when comparing the cryo-EM structures of the protein in its open and closed states. The flow of such changes along chains of residues throughout the protein in response to disease associated point mutations or experimental manoeuvres can now be followed from the site of the affected residue on the huge cytoplasmic domain of the protein through domains deep within the protein to the ion channel pore located within the distant membrane domains, for example (Iyer et al. 2020; Woll et al. 2021). These advances have finally allowed an answer to the old conundrum of how can binding sites on the surface of the cytoplasmic extremities of the giant RyR1 molecule influence the opening of the ion channel located within the SR membrane tens of angstroms away from the mutation or drug binding sites.

### The voltage sensor in the junctional T-tubule membrane

During the late 1960’s and early 1970’s there was much debate about whether the molecule that sensed changes in the voltage across the T-tubule membrane was in fact a protein and debate about the molecular mechanism of voltage-sensing. A pivotal discovery in excitable membrane physiology at that time was a minute signal seen using voltage clamp electrophysiology and seen clearly only in the absence of all ionic currents. The signal, colloquially known as “charge movement”, was thought to reflect movement of dipoles in the membrane spanning parts of voltage-sensing proteins to initiate voltage-sensitive events such as ionic currents or indeed EC coupling (Armstrong and Bezanilla 1974; Catterall et al. 2017).

The correlation between charge movements and voltage-dependence of EC coupling (Dulhunty and Gage 1983) as well as the sensitivity of EC coupling to the drug nifedipine (Lamb 1987; Rios and Brum 1987; Dulhunty and Gage 1988) provided compelling evidence that the voltage-sensing molecule was Ca_V_1.1, at that time known as the DHPR or L-type Ca^2+^ channel and subsequently reclassified as Ca_V_1.1. The molecular identity of the voltage sensor was finally confirmed during the 1990’s with the observation that EC coupling was restored when the recombinant Ca_V_1.1 was expressed in Ca_V_1.1-null myotubes (Tanabe et al. 1988; Takekura et al. 1994). Over the next 40 years many studies delved into the complexities of the structure and function of voltage sensor, continuing to use voltage clamp electrophysiology along with molecular biology and site directed mutagenesis to determine specific residues in Ca_V_1.1 that are essential for EC coupling and the residues that sense voltage to initiate EC coupling as opposed to those that open the ion channel pore (Pelizzari et al. 2024).

**Fig. 4:**
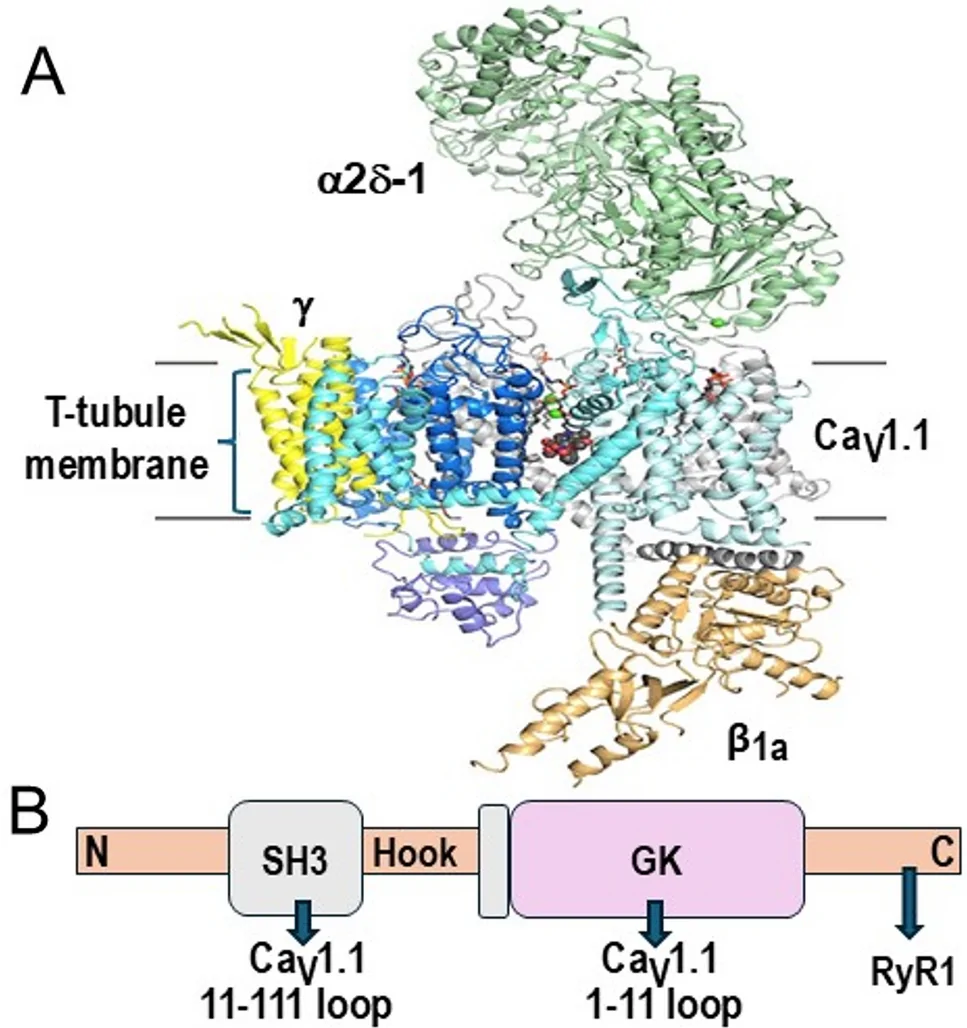
Cryo-EM structure of Ca_V_1.1 with associated subunits and the domain architecture of the β_1a_ subunit. **A** The structure of Ca_V_1.1 is coloured in shades of blue and is shown embedded in the T-tubule membrane. Ca_V_1.1 is shown in a complex with the β_1a_ subunit (brown) in the cytoplasm, the γ subunit (yellow) in the T-tubule membrane and the α2δ-1 subunit (green) in the lumen of the T-tubule. Modified from (Zhao et al. 2019). (**B**) The structural domains of β_1a_. The split SH3 domain (grey) is located towards the N terminus of β_1a_ and the guanylate kinase (GK) domain (pink) is located towards the C terminus. Modified from (Karunasekara et al. 2025). The arrows in **B** indicate reported binding interactions of the SH3 domain of β_1a_ with the Ca_V1.1_ II-III loop and the GK domain of β_1a_ binding to the Ca_V_1.1 I-II loop as labelled. The possibility of C-terminal tail of β_1a_ binding to RyR1 is also indicated (Rebbeck et al. 2011)

Structurally, Ca_V_1.1 is a typical voltage gated cation channel, composed of four membrane spanning domains or repeats, separated by loops throughout the cytoplasmic solution (Fig. 4A). The four membrane spanning domains surround the central Ca^2+^ ion channel pore. Each membrane spanning domain contains four transmembrane helices and the fourth (S4) helix of each domain contains charged residues that could move with changes in the electrical field within the membrane and generate “charge movement” (Catterall et al. 2017). A significant question has been whether one of the individual voltage sensing domains gates the ion channel current and a different domain initiates EC coupling, or do the same domains regulate both processes? Recent evidence indicates that voltage-sensing domain III exclusively controls skeletal muscle EC coupling (Pelizzari et al. 2024; Angelini et al. 2025).

The exquisite structural detail of the voltage-sensing Ca_V_1.1 bound to the β_1a_, the α2δ-1 and the γ subunits was revealed in a subsequent cryo-EM analysis (Fig. 4) (Zhao et al. 2019; Wu et al. 2016). Interestingly, analysis of the cryo-EM data yielded two classes of structure one with a prominent displacement of β_1a_ with adjacent elements of Ca_V_1.1 (Wu et al. 2016). The existence of two structures leads to key questions including whether this molecular displacement is a part of the mechanism underpinning EC coupling? A pressing question has been which of the cytoplasmic loops contain sequences that bind to other proteins within the junctional gap and potentially form a part of the EC coupling pathway across the junction? The C region of the Ca_V_1.1 II-III loop (residues 720–765) was found to be crucial for skeletal EC coupling (Nakai et al. 1998; Grabner et al. 1999; Wilkens et al. 2001). However, despite determined efforts in time and resources, evidence for direct binding of the II-III loop in full length Ca_V_1.1 to the full length RyR1 has not been demonstrated. Therefore, suggesting that the relevant interaction of the II-III loop could occur through intermediatory proteins. Indeed, it has now been shown that different regions of the II-III loop bind to the β_1a_ subunit and to STAC3. STAC3 also binds to residues in the C-terminus of Ca_V_1.1 suggesting the possibility that multiple binding interactions between the core proteins could contribute to EC coupling (Tuinte et al. 2025; Karunasekara et al. 2025) as discussed in more detail below.

### Proteins within the junctional space that link CaV1.1 to the RyR1

It was initially thought that EC coupling depended on a signal passing directly from the CaV1.1 to RyR1. However, despite exhaustive efforts it became apparent that one or more cytoplasmic proteins in the junctional space *must* interact in some way with Ca_V_1.1 and RyR1 to transmit the signal. Such a massive macromolecular complex would extend from the extracellular solution within the T-tubule lumen, cross the junctional space and terminate in the lumen of the SR. One of the first cytosolic proteins to explored was the β_1a_ subunit of Ca_V_1.1 (Fig. 4).

#### The β_1a_ subunit of CaV1.1

β_1a_ has diverse roles in both triad formation and EC coupling. It was also shown to target Ca_V_1.1 to the T-tubule membrane and to modulate the ion channel function of Ca_V_1.1 (Gerster et al. 1999; Bichet et al. 2000) and was a logical contender for a possible role in mechanical EC coupling. It was found to modulate mechanical coupling via its C-terminal tail (Ahern et al. 2003; Cheng et al. 2005) and was later confirmed to be one of the four core EC coupling proteins (Perni et al. 2017). Freeze fracture studies revealed that the C-terminal tail of β_1a_ is essential for DHPR tetrad formation (Dayal et al. 2022). It is perhaps significant that a peptide corresponding to the proximal C-terminus of the β_1a_ subunit, binds directly to RyR1 (Rebbeck et al. 2011) and modulates the strength of EC coupling (Hernandez-Ochoa et al. 2014). We recently reported that an interaction between the Ca_V_1.1 II-III loop and the β_1a_ subunit impacts EC coupling (Karunasekara et al. 2025). The β_1a_ subunit also binds to a region on the I-II loop of Ca_V_1.1 known as the alpha interaction domain (AID), while conversely AID binds to a hydrophobic groove in a conserved GK region of β_1a_ proximal to its C-terminal tail (Buraei and Yang 2010). It is relevant in considering the various pathways that EC coupling might travel across the junctional gap (Fig. 6 below), that AID covers only a small part of the surface of β_1a_ so that much of the surface remains available for interactions with other regions of Ca_V_1.1 or with other proteins in the junctional space (Buraei and Yang 2010).

#### STAC3

**Fig.5:**
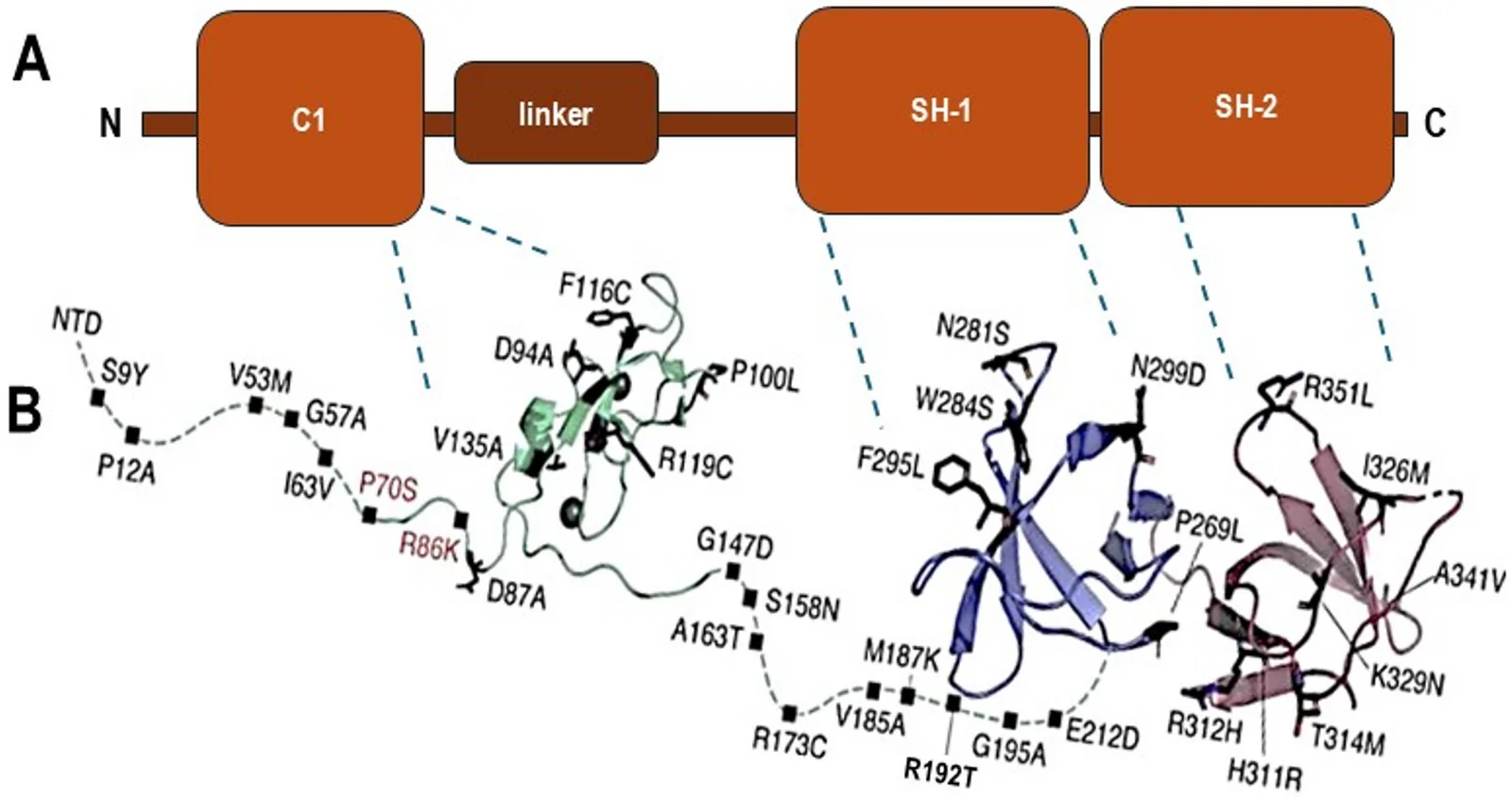
Domain structure and 3D arrangement of STAC3. **A** Illustrates the relative position the C-terminal SH3-1 and SH3-2 domains that contribute to EC coupling as well as the C1 domain in the N-terminal region that is not implicated in EC coupling, although involved in protein–protein interactions that are important for STAC3 incorporation in the triads. The position of linker region between the C1 and SH3 domains is also indicated. Modified from (Flucher and Campiglio 2019). (**B**) An unfolded representation of the STAC3 protein showing the NMR ensemble structure for the STAC3 C1 domain and the X-ray crystal structure of the tandem SH3 domains of STAC3. Each domain is composed of a five-stranded antiparallel beta sheet (Rufenach and Van Petegem 2021). The locations of several reported sequence variants in STAC3, possibly linked to disease, are labelled with black text and potential disease-associated variants in STAC2 are shown labelled with brown text. The broken lines between **A** and **B** indicate structures in **B** that are associated with the domains in **A**. (**B**) and (**C**) Modified from (Rufenach and Van Petegem 2021)

The last of the core EC coupling proteins to be identified has been the adaptor protein STAC3. STAC3 has three well defined structural domains. The C1 domain of STAC3 resides in the N-terminal part of the protein and the SH3-1 and SH3-2 domains are in the C-terminal half of STAC3 as shown in Fig. 5A. It is relevant that both C1 domains and SH3 domains are well recognised for their roles in a variety protein–protein interactions (Rufenach and Van Petegem 2021). The 3-dimensional structure of the tandem SH3 domains has been determined and is shown in Fig. 5B while the NMR structure of C1 has been deposited in the PDB (2DB6) (Rufenach and Van Petegem 2021). Therefore, in the context of its role as an adaptor protein, STAC3 is ideally structured to take part in the protein interactions that contribute to EC coupling. STAC3 was first implicated in skeletal EC coupling when a mutation in the protein was identified as the cause of the congenital Native American Myopathy (NAM) (Horstick et al. 2013; Polster et al. 2016). As mentioned above (Fig. 2), STAC3 binds to two sites on Ca_V_1.1. At the first site the C-terminal SH3-1 domain of STAC3 binds to the II-III loop of Ca_V_1.1 which is essential for EC coupling (Nakai et al. 1998). The second site involves the C1 and linker of STAC3 binding to the EF hands and IQ domains proximal to the C-terminus of Ca_V_1.1 (Fig. 5) (Tuinte et al. 2025). As with β_1a_, STAC3 has roles in both EC coupling and in the organisation of the proteins within the triad junction (Tuinte et al. 2025). The fact that STAC3 is localised in the triads in the absence of Ca_V_1.1 indicates that there are additional STAC3 binding sites in the triad junction as well as those on Ca_V_1.1 and potentially on β_1a_ (Campiglio et al. 2018). STAC3 is also involved in tetrad stabilisation (Perni et al. 2017). Finally, STAC3 fine tunes the characteristics of the Ca^2+^ current through Ca_V_1.1, in addition to its fundamental roles in supporting skeletal EC coupling and in targeting Ca_V_1.1 to the triad junction (Tuinte et al. 2022).

A highly significant observation in understanding the various interactions in the transmission of the EC coupling signal through the triad junction has been the survival of a patient lacking the SH3-1 and SH3-2 domains of STAC3 (Tuinte et al. 2025; Karunasekara et al. 2025). This indicates that there are likely to be additional pathways that can support a weak component of skeletal EC coupling as discussed in the following section addressing potential coupling pathways. In conclusion, STAC3 plays a fundamental role in skeletal muscle function even though some of its binding sites may not yet have been identified and the molecular mechanisms underlying its contribution to EC coupling remain to be fully defined.

### Consideration of EC coupling pathways between Ca_V_1.1 and RyR1

**Fig. 6:**
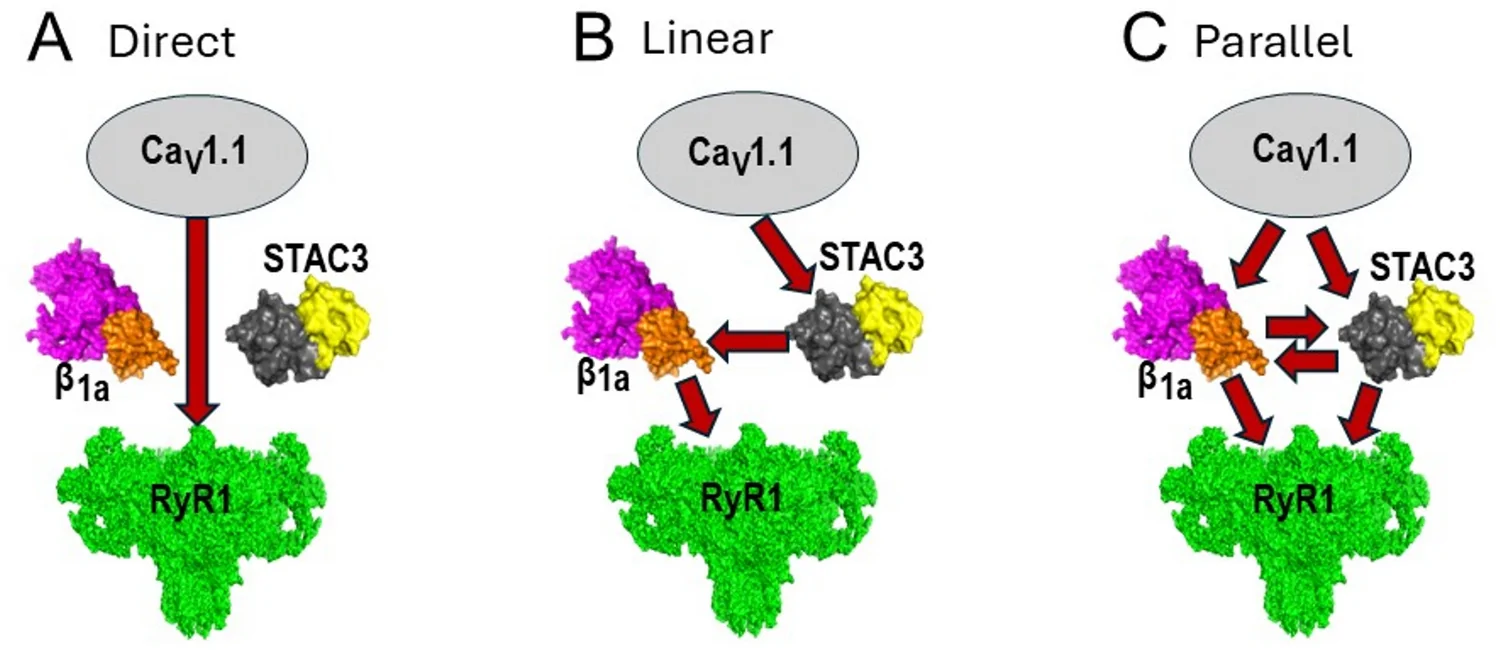
Examples of potential pathways between Ca_V_1.1 and RyR1. **A** Direct connection between Ca_V_1.1 and RyR1. **B** A potential linear path from Ca_V_1.1 to STAC3 then to β1a and finally to RyR1. The potential linear path could equally travel from Ca_V_1.1 to β1a then to STAC3 and to RyR1. **C** Parallel paths that could include any (or all) combinations of connections between Ca_V_1.1 and RyR1 that include β_1a_ and STAC3. The connections in **B** and **C** illustrate possible types of communication rather than a specific pathway as there are many possible alternative combinations. Similarly binding sites on the proteins are not specifically indicated because, although interaction sites have been identified as described Table 1, additional sites have not been excluded

A study reporting the presence of residual EC coupling exists in the absence of the STAC3 SH3 domains prompted speculation about the possibility of parallel pathways for EC coupling transmission across the triad junction involving the core EC coupling proteins (Tuinte et al. 2025; Karunasekara et al. 2025). Three general models that would allow Ca_V_1.1 to communicate with RyR1 are illustrated in Fig. 6. The first model is simply a direct communication between Ca_V_1.1 and RyR1 (Fig. 6A). Direct communication is conceivable because the dimensions of the junction gap (approximate 10 nm wide) would accommodate cytoplasmic domains of both proteins. Direct binding of the two ion channels was initially assumed to be the transmission mechanism however early experiments failed to demonstrate binding between Ca_V_1.1 and RyR1 in either protein complexes isolated from skeletal muscle or between the isolated proteins (Rebbeck et al. 2014; Bannister 2016; Meissner 2017; Flucher and Campiglio 2019).

The second model shown in Fig. 6B evolved with the discovery that EC coupling depended critically on the presence of β_1a_ and STAC3 ((Perni et al. 2017). Since STAC3 binding to RyR1 has yet to be demonstrated, the model in Fig. 6B shows a linear pathway across the junction that passes from Ca_V_1.1, through STAC3 and then through β_1a_ to RyR1. Alternatives clearly exist, although not shown in Fig. 6B. Additional interactions between the proteins may well emerge if additional binding sites between the proteins are discovered. A possible alternative is the signal passing from Ca_V_1.1 via β_1a_ to RyR1.

The third type of model outlined in Fig. 6C illustrates parallel pathways across the triad junction. The possibility of parallel signalling pathways is attractive because it would include structural and functional interactions observed between the core proteins that are difficult to envisage with a linear model, for example (Sheridan et al. 2006). Parallel pathways might well include a strong pathway between the II-III loop and STAC3 and weaker pathways involving for example β_1a_. In a broader context, the existence of parallel pathways would provide a degree of redundancy in EC coupling to ensure survival if one path was compromised by a loss-of-function mutation, as with the patient living with a STAC3 variant that lacks the tandem SH3 domain that has been assumed to be critical for EC coupling (Tuinte et al. 2025). To reiterate, the SH3-1 domain interacts with a 47-residue segment of the Ca_V_1.1 II-III loop which includes II-III loop C region resides that are essential for EC coupling (Nakai et al. 1998). The II-III loop also binds to the SH3 domain β_1a_, so that a pathway to RyR1 through β_1a_, would remain if the STAC3 interaction with either RyR1 or Ca_V_1.1 II-III loop was interrupted (Fig. 6C)**.** The problem with this model is again the fact that STAC3 binding to RyR1 has not yet been demonstrated. However, the field is evolving and binding interactions between RyR1 and STAC3 may well be demonstrated in the future. With both the linear and parallel models, the number of different possible combinations that could form a pathway is limited only by our imagination.

### Known and unknown facets of skeletal muscle EC coupling.

Many facts about the overall process of EC coupling in skeletal muscle are now known, although the fundamental molecular interactions that underlie the mechanism remain undetermined. To summarise, the established facts are that EC coupling is localised to the junction between the T-tubule and SR membranes and that the core proteins that support transmission of the signal across the junction have been identified. Binding sites have been identified between Ca_V_1.1 and its β_1a_ subunit, between Ca_V_1.1 and STAC3 and between β1a and RyR1 (Table 1). Direct binding has not been shown between RyR1 and Ca_V_1.1, between RyR1 and STAC3 or between STAC3 and β_1a_. It is important to note that most experiments exploring binding sites have been performed with isolated proteins. None of the binding sites have been confirmed in intact living mammalian skeletal muscle fibres. However, Zebrafish have provided a useful alternative in addition to various preparation of myocytes and myotubes as well as muscle-derived cell lines. The recent in-situ cryo-ET experiments using snap frozen functioning mouse EDL fibres provide an interesting technical development (Xu et al. 2024). The major unknowns surround the inter-molecular and-intra molecular mechanisms that proceed during EC coupling and indeed the molecular pathway or pathways that are followed across the triad junction.

**Table 1:**
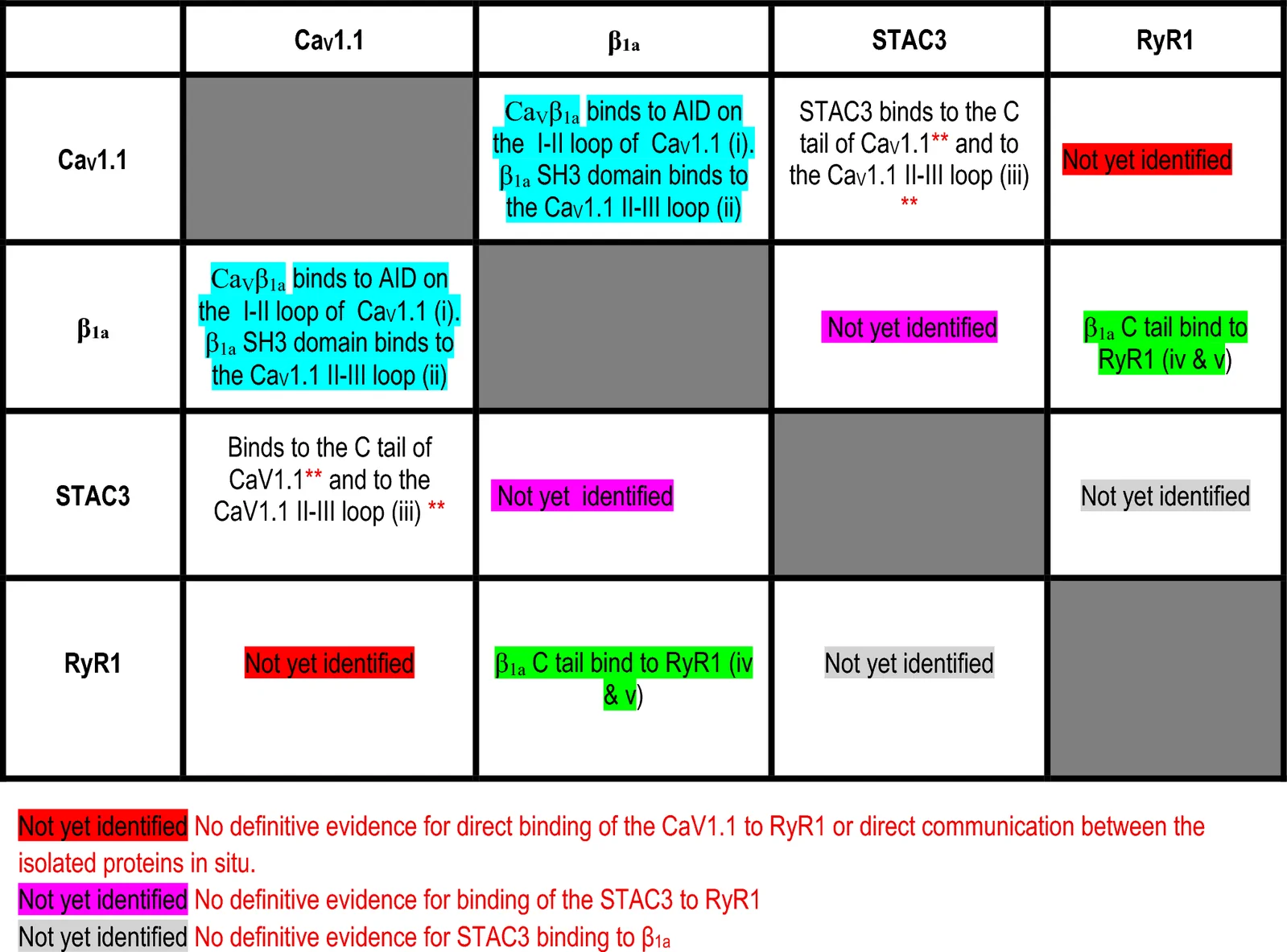
Matrix summery of interactions that have been identified between the four EC coupling proteins

Interactions between the intact proteins and peptide segments of the proteins are included. Supporting publications are indicated by numbers (i) to (v): (i) (Buraei and Yang 2010), (ii) (Karunasekara et al. 2025), (iii) (Tuinte et al. 2025), (v) (Cheng et al. 2005), (vi) (Rebbeck et al. 2011), ** Direct impact EC coupling Red asterisks ** indicate that effects of interaction on EC coupling have been observed. Note that knock out of any of the four core proteins abolishes EC coupling (Perni et al. 2017). Highlight colour is included to help visualise the matrix

### Future directions. Positioning the main players together in the triad junctional space

**Fig. 7:**
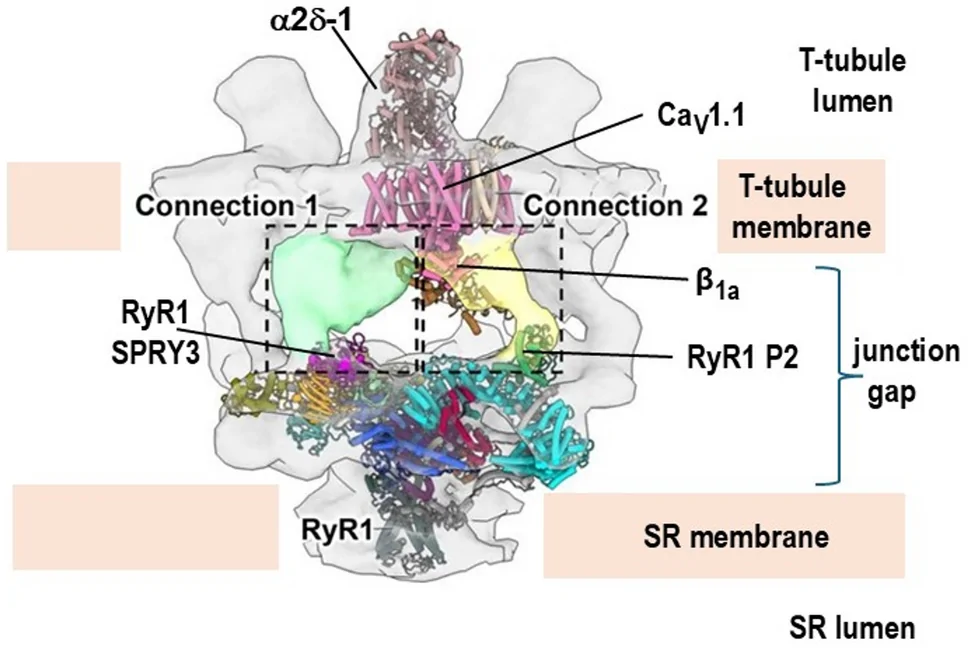
In-situ structure of the RyR1/Ca_V_1.1 super-complex in a native triad junction. The images were obtained using in-situ cryo-ET techniques applied to triad junctions isolated from snap frozen living intact skeletal muscle fibres prepared from the extensor digitorum longus (EDL) muscle of 2-to3-week-old mice. The image shows densities indicating the two connections between RyR1 and the Ca_V_1.1/β_1a_/α2δ-1 protein complex which are displayed in green (Connection 1) and yellow (Connection 2) with the complex in each case contained within the surrounding box. Connection 1 lies between the Ca_V_1.1 complex and the SPRY3 domain of RyR1. SPRY domains are generally known to provide binding sites for regulatory proteins (Tae et al. 2009; Peralvarez-Marin et al. 2011). Connection 2 lies between Ca_V_1.1/ β_1a_ regions and RyR1 P2 and HD2 domains. The HD2 domain is located in the vicinity of contact points between neighbouring RyR1 tetramers (Xu et al. 2024). Therefore Connection 2 may be involved coupled gating between RyRs and thus possibly in the spread of EC coupling signals from CaV1.1-coupled RyR1 associated with tetrads to Ca_V_1.1-uncoupled RyR1, discussed earlier in this communication. Modified from (Xu et al. 2024)

A major obstacle in the quest to discover how Ca_V_1.1 communicates with RyR1 has been an inability to solve the structure of the two ion channels in-situ. A breakthrough in sample preparation was achieved using a cryo-focused ion beam technique that allowed an in-situ cryo-ET study of intact mammalian triad junctions (Xu et al. 2024). This technique although in its infancy, has already revealed the spatial relationship between the two ion channels and indicated regions of two interaction sites between the Ca_V_1.1 β_1a_ complex with RyR1 (Fig. 7). Given that only low-resolution $(16.7\mathop A\limits^{^\circ } - RyR1,{\text{ }}33\mathop A\limits^{^\circ } - CaV1.1)$ images have so far been obtained it is significant that the two interacting sites have been identified. The two sites correspond to the SPRY3 and HD2 regions of RyR1. More detailed information about the residues supporting the interactions within the two sites and how the smaller STAC3 and β_1a_ are involved will become available with advances in in-situ cryo-ET techniques. It remains to be determined whether these two sites are directly or indirectly involved in EC coupling?

Overall, the evolving techniques of in-situ cryo-ET are varied and expanding and likely to answer many of the remaining questions. However, there are some limitations with this technology, most of which involve insufficient resolution to map key atomic interactions. This is particularly important where high-resolution structural interactions must be resolved under resting and depolarisation-activated conditions to fully understand the molecular mechanisms. Another limitation is that the in-situ cryo-ET images provide a static snapshot of a dynamic process, so they do not reveal the way binding interactions might change on a microsecond time scale during the dynamic EC coupling process. Until future technological advances are accessible, researchers will have to continue exploring the mechanisms using a variety of complimentary techniques. The techniques include lower resolution structures in combination with high resolution structures derived from cryo-EM, Nuclear Magnetic resonance (NMR) and X-ray crystal structures (Xu et al. 2024) as well as other techniques including Alpha Fold-Multimer predictions, in fibre FRET-based reporters (Robinson et al. 2026) and molecular dynamics simulations (Lile et al. 2026). All such techniques will contribute to our ability to tackle the challenges of mapping the real time structural fluctuations that contribute to skeletal EC coupling. Among other benefits mentioned in the Concluding Comments below, the results from future high resolution in-situ cryo-ET procedures will allow researchers to further explore evolving evidence regarding parallel pathways for EC coupling (Tuinte et al. 2025; Karunasekara et al. 2025).

### Concluding comments

The elusive and complex mechanism of skeletal muscle EC coupling has provided a major challenge for researchers over the past decades. Despite the wealth of knowledge accumulated over this time, we still do not understand the detailed molecular changes that *must* take place to transmit the EC coupling signal across the triad junction. A pivotal finding in attempts to define the molecular pathways for EC coupling across the triad junction has been the survival of the patient with a deletion mutation in the STAC domains that were previously thought to be *essential* for EC coupling (Tuinte et al. 2025). Generally, conceptual leaps in understanding skeletal muscle EC coupling have followed major technical advances. It is likely that in future years further breakthroughs in the field will continue to be dictated by technical advances in structural techniques that allow increased structural resolution. In-situ cryo-ET techniques are already progressing towards greater image resolution that will solve the three-dimensional arrangement of the core proteins within the junctional gap (Xu et al. 2024). In the meantime, the recent developments in static cryo-ET techniques will be complemented by combining data with results from high resolution cryo-EM (Xu et al. 2024). Further refinements by combining data with AlphaFold-Multimer predictions of the tetrad complex, molecular dynamics simulations, and the FRET-based reporters will help to develop models that probe the structural dynamics within the triadic junctional region during EC coupling. Further into the future, the development of techniques may allow visualisation of the atomic changes within and between the core proteins in living muscle fibres during EC coupling on the microsecond time scale of the signal transmission across the triad junction. With an increase in this depth of knowledge the molecular changes in EC coupling that cause debilitating muscle weakness will be better understood and treatments developed to strengthen Ca^2+^ release and muscle output that are appropriate for different underlying defects.

## Data Availability

No new data is presented in the manuscript.

## Abbreviations

AID: Alpha interaction domain
ATP: Adenosine triphosphate
α2δ-1: Delta subunit of Ca_V_1.1
apo-CaM: Calcium-free calmodulin
β_1a_: Beta subunit of Ca_V_1.1
C1: Protein domains recognised for a role in protein–protein interactions
CaM: Calmodulin
Ca_V_: A class of dihydropyridine-sensitive voltage-dependent cation channels
Ca_V_1.1: Voltage-dependent cation channel expressed in skeletal muscle, (Footnote 1: Voltage-sensitive Ca^2+^ channel proteins in skeletal (Ca_V_1.1) and cardiac (Ca_V_1.2) muscle are variously referred to in the literature as DHPR, L-type Ca^2+^ channels or α subunit of the DHPR. The name DHPR is also used to describe the Ca_V_ protein complexed with its, β. δ and γ gamma subunits. This terminology is technically incorrect because the DHP binding site is located only in the Ca_V_ protein and does not include its subunits (Tikhonov DB, Zhorov BS (2023) Mechanisms of dihydropyridine agonists and antagonists in view of cryo-EM structures of calcium and sodium channels. J Gen Physiol 155https://doi.org/10.1085/jgp.202313418)
Ca_V_1.2: Voltage-dependent cation channel expressed in the heart, (See footnote 1)
Ca_V_1.1 I-II loop: Cytoplasmic loop linking 1^st^ and 2^nd^ transmembrane repeats of Ca_V_1.1
Ca_V_1.1 II-III loop: Cytoplasmic loop linking 2^nd^ and 3^rd^ transmembrane repeats of Ca_V_1.1
CNS: Central nervous system
Cryo-EM: Cryo-electron microscopy
Cryo-ET: Cryo-electron tomography
Diad: Junction between the surface or T-tubule membrane and one terminal cisterna of the SR
DHPR: Dihydropyridine receptor (See footnote 1)
EF Hand: A helix–loop–helix structural motif found in many protein calcium-binding sites
EC coupling: Excitation–contraction coupling
EM: Electron microscopy
FKBP12: FK506 binding protein closely associated with RyR1
γ: Gamma subunit of Ca_V_1.1
GK domain: Guanylate kinase domain of β_1a_
HD1: A structural domain in the centre of the cytoplasmic mass of RyR1 facing the T-tubule membrane
HD2: A structure in the centre of the cytoplasmic mass of RyR1 facing the SR membrane and separated from the HD1 domain by the P2 domain
Homotetramer: A functional protein formed by association four identical monomers
IQ domains: IQ domains interact with EF-hand motifs forming likely targets for calmodulin interactions with RyR1 which modify EC coupling in skeletal muscle
P2: A structural domain of RyR2 separating the HD1 and HD2 domains, close to HD1 and extending towards the T-tubule membrane
JP2: Junctophilin 2
NMR: Nuclear magnetic ressonance
RyR: Ryanodine receptor
RyR1: Ryanodine receptor type 1, or skeletal RyR
RyR2: Ryanodine receptor type 2, or cardiac RyR
RyR3: Ryanodine receptor type 3, sometimes referred to as brain RyR
SPRY: A protein interaction module. There are 3 SPRY domains in RyR1
SH3 domains: Ubiquitous interactive proline rich domains supporting protein–protein interactions
SR: Sarcoplasmic reticulum
STAC3: A muscle-specific adaptor protein
TC: Terminal cisternae
Tetramer: A functional protein formed by the close association of four monomers
Tetrad: A cluster of four Ca_V_1.1 particles within a 30 nm x 30 nm square area in freeze fracture replicas of the surface membrane of muscle-derived cells
Triad: Junction between T-tubule membrane and two terminal cisternae expansions of the SR, one on either side of the T-tubule
T-tubule: Transverse tubule
IQ domains: Domains that interact with EF-hand motifs forming likely targets for calmodulin interactions with RyR1 which modify EC coupling in skeletal muscle
